# Supplementary material for: Chronic Voluntary Morphine Intake Is Associated with Changes in Brain Structures Involved in Drug Dependence in a Rat Model of Polydrug Use
Source: Int J Mol Sci. 2023 Dec 3;24(23):17081. doi: 10.3390/ijms242317081 (PMC10707256; doi:10.3390/ijms242317081)
Supplement: Supplementary file 1 [file ijms-24-17081-s001.zip › ijms-2729171-Supplementary.pdf]

International Journal of *Molecular Sciences*

MDPI

Supplementary Material

Chronic voluntary morphine intake is associated with changes in brain structures involved in drug dependence in a rat model of polydrug use

María Elena Quintanilla; Paola Morales, Daniela Santapau, Alba Ávila, Carolina Ponce, Pablo Berrios-Cárcamo, Belén Olivares, Javiera Gallardo, Marcelo Ezquer, Mario Herrera-Marschitz, Yedy Israel, Fernando Ezquer.

Supplementary Figure S1

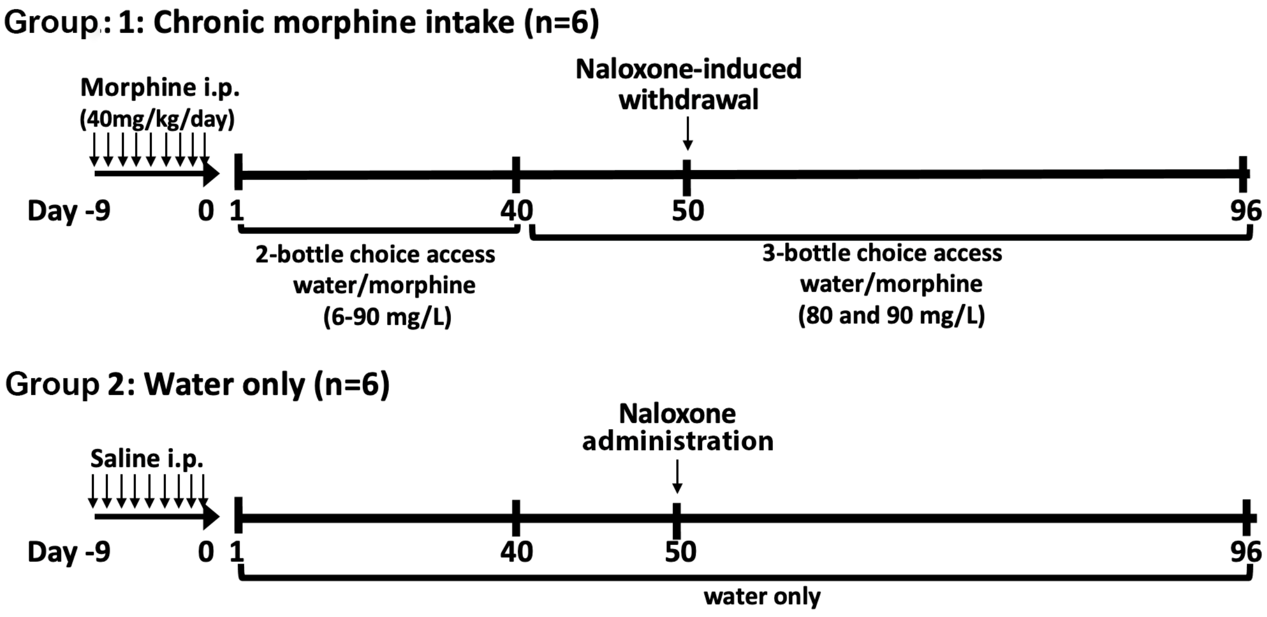

Supplementary Figure S1: Experimental Design. Fifty-day-old female UChB rats were administered a daily dose of 40 mg/kg i.p. of morphine hydrochloride for 9 consecutive days (days -9 to 0 Group 1). Animals in group 2 (control) were administered saline on days -9 to 0. On day 1, individual cages of animals in Group 1 were fitted with a second drinking bottle containing a morphine solution at increasing concentrations on successive days (6 to 90 mg/L on days 1 to 40). From days 41 to 96, rats were concurrently provided with free choice access among three bottles: one containing water and the other two with 80 and 90 mg/L morphine sulfate solutions. Control animals (Group 2) drank only water for 96 days. On day 50, animals were injected with one dose (5 mg/kg, i.p.) of the opioid antagonist naloxone to determine whether they were morphine-dependent.

Supplementary Figure S2

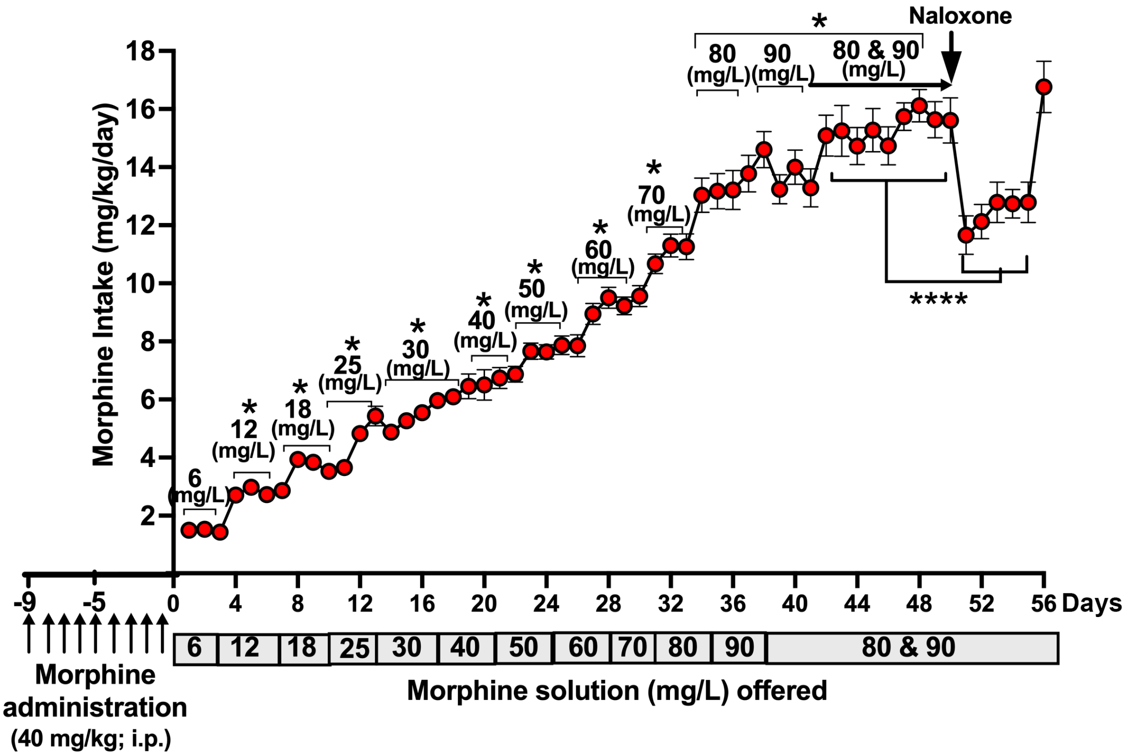

Supplementary Figure S2. Increase in voluntary morphine consumption following the repeated intraperitoneal administration of morphine (40 mg/kg/day) and subsequent free access to a morphine solution of escalating concentrations and water. Reduction of morphine intake following administration of one dose (5 mg/k/day, ip) of naloxone. The figure shows the morphine consumption, expressed as mg morphine sulfate consumed per kilogram of body weight per day (mean ± SEM), of rats provided with free choice between a morphine solution of increasing concentration and water 24 hours/day (n=6). Arrows (days -9 to 0) indicate intraperitoneal administration of morphine hydrochloride. One-way ANOVA of all voluntary morphine intake data shown in the figure indicated a significant effect on morphine intake of the concentration of the morphine solution offered to rats ($F_{concentration\ (11,116)}$= 181.2, $p < 0.0001$). Tukey's post hoc test revealed that each increase in morphine concentration in the range of 6 mg/L to 90 mg/L resulted in a significant increase (*p<0.0001) in daily morphine intake. On day 41 and onwards, rats were concurrently provided with free choice access to three bottles: one with water and the other two with different concentrations of morphine sulfate (80 and 90 mg/L) that were kept constant for 55 days. On day 42, the animals reached an average steady state of morphine consumption of 15.3 ± 0.22 mg/kg/day (mean ± SEM). On day 50, to determine whether this level of morphine intake induced morphine dependence, they were injected with one dose (5 mg/kg, i.p.) of naloxone. Analysis of the effect of naloxone on morphine intake indicated that naloxone administration reduced morphine consumption by 20% for five days (**** p < 0.0001 means significant difference between the average (mean ± SEM) of the five days pooled data after naloxone administration [12.8 ± 0.29 (mean ± SEM), days 51 to 55] compared with the average (mean ± SEM) of the 9 days pooled data before naloxone administration [15.3 ± 0.22 (mean ± SEM), days 42 to 50).

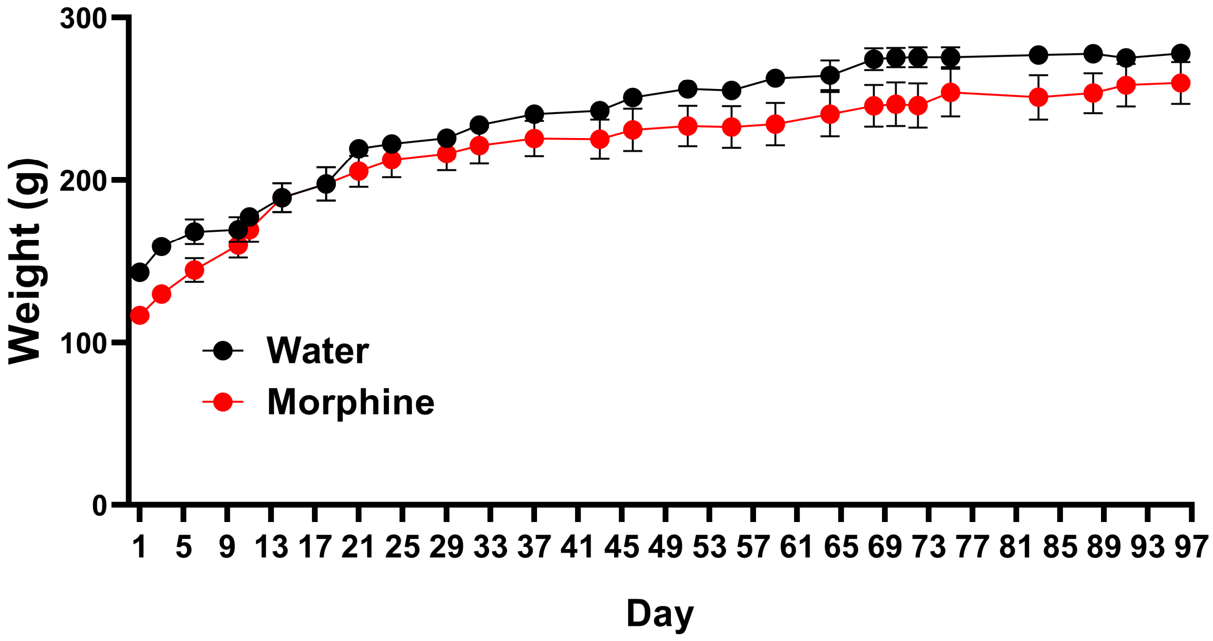

Supplementary Figure S3: Body weight of animals consuming voluntary morphine or only water. Body weight was measured every 4 days. Two-way ANOVA revealed no effect of treatment (F_treatment(1,10) = 2.60, $p < 0.13$; N.S.), although the day had an effect (F_day(2.883,28.83) = 237.37, $p < 0.0001$). Sidak post hoc analysis indicated no significant effect between the two experimental groups ($p > 0.05$), n=6.
